# Epidemiology and *emm* types among group A streptococcal pharyngitis in Finland: a prospective laboratory-based study

**DOI:** 10.1007/s10096-023-04714-6

**Published:** 2023-11-27

**Authors:** Mirva Virolainen, Kirsi Gröndahl-Yli-Hannuksela, Kaisu Rantakokko-Jalava, Tapio Seiskari, Emilia Lönnqvist, Terhi Kolari, Tiia Rissanen, Hanne-Leena Hyyryläinen, DICAR study group, Ville Kailankangas, Jaana Syrjänen, Johanna Vilhonen, Jarmo Oksi, Risto Vuento, Jaana Vuopio

**Affiliations:** 1 Institute of Biomedicine, University of Turku, Kiinamyllynkatu 10, 20520 Turku, Finland; 2 Turku University Hospital, Clinical Microbiology, Turku, Finland; 3 Fimlab laboratories, Tampere, Finland; 4 Department of Biostatistics, University of Turku and Turku University Hospital, Turku, Finland; 5 Finnish Institute for Health and Welfare, Helsinki, Finland

**Keywords:** Group A streptococcus, *S. pyogenes*, GAS, Pharyngitis, *emm* type, Seasonality, Epidemiology

## Abstract

**Purpose:**

*Streptococcus pyogenes* (mostly termed group A Streptococcus - GAS) is the most important bacterial causative of pharyngitis. However, epidemiology of GAS pharyngitis is not widely established. This study describes GAS pharyngitis cases and *emm*-type distribution in a prospective study covering over 2 years in two Hospital Districts in Finland.

**Methods:**

A prospective, systematic collection of GAS pharyngitis isolates was conducted between March 2018 and December 2020 in two large Hospital Districts in Finland. Patient characteristics (age, gender) were included if available. All GAS isolates collected were *emm* typed.

**Results:**

Altogether 1320 GAS pharyngitis strains were collected, 904 in the Hospital District 1 (HD1) and 416 in Hospital District 2 (HD2). In HD1, age and gender data were available. Females were overrepresented (58% of all cases). In addition, the age and gender distributions were noted to be significantly different (*p* < 0.0001) with females having a more uniform distribution until age of 40. *emm*28 was common among the age group of 20–29-year-olds and *emm*89 in children under 10 years of age, respectively. In HD1, most of the isolates were collected during winter and autumn months. Significant differences by season in the frequency of *emm*12, *emm*89, *emm*75 and group of “others” were observed.

**Conclusion:**

Age distribution among GAS pharyngitis cases was significantly different between genders (*p* < 0.0001). In addition, age group specific and seasonal variations in *emm* GAS types causing the disease were observed. These findings warrant further investigation, especially for understanding population-based spread of GAS even in more detail.

**Supplementary Information:**

The online version contains supplementary material available at 10.1007/s10096-023-04714-6.

## Introduction

Group A Streptococcus (*Streptococcus pyogenes*, GAS) is an important human pathogen causing infections from mild pharyngitis to life-threatening invasive infections (iGAS) [1–3]. Pharyngitis is the most common disease manifestation of GAS. In 5–15% of adults and 15–35% of children with pharyngitis, GAS is found as the causative agent [1–4]. Asymptomatic throat carriage is also recognized especially in young children [5]. Since late 2022, several European countries have reported increased numbers of GAS infections, especially scarlet fever and iGAS infections in children [6–8].

M-protein is the most important virulence factor of GAS. *Emm* typing is based on sequencing of the hypervariable region of *emm* gene that codes the M-protein. There are currently over 260 different *emm* types recognized [9]. Same *emm* types may cause both invasive and mild infections, and associations between specific *emm* types and certain infection foci have been reported [3, 10]. Currently, there is no vaccine available against GAS. However, there are several vaccine candidates in clinical trials [11]. Reports on epidemiology and distribution of *emm* types and among iGAS are available [12], but those on GAS pharyngitis are scarcer [3].

Here we describe the results from a prospective over 2-year study on epidemiology of GAS pharyngitis in two large hospital districts in Finland. Our results show a significant difference in the prevalence of GAS pharyngitis between different age groups and gender. In addition, a clear pattern of seasonal variation in *emm*-type distribution was observed.

## Methods

### Study settings

GAS throat cultures were collected from two clinical microbiology laboratories in Finland: Turku University Hospital, Clinical microbiology laboratory in Hospital district of Southwest Finland (hereafter HD1), serving a population of 470,000 and Fimlab laboratories in Pirkanmaa Hospital district (hereafter HD2), serving a population of 520,000. At the time when this study was conducted, the Finnish Current Care Guidelines on diagnostics of acute pharyngitis recommended a throat culture to be performed especially if GAS infection and subsequent antimicrobial treatment was considered. This practise was commonly followed.

In HD1, the clinical laboratory randomly selected 10 MALDI-TOF confirmed *Streptococcus pyogenes* cultures from their routine pharyngitis diagnostics to be included in this study. Isolates were collected weekly for a 32-month period (March 2018–December 2020). Collection halted for 6 weeks (16.3.–26.4.2020) due to the COVID-19 pandemic. The culture plates were transferred to the University of Turku, for analysis and storage. If more than 10 cultures were delivered, all were included to have a good presentation of the circulating isolates. The isolation date and age and gender of the patients were recorded. The data was anonymized, and only arbitrary study codes were used.

For comparison, simultaneous collection of *S. pyogenes* pharyngitis isolates from HD2 was conducted. Similarly, to HD1, MALDI-TOF confirmed *S. pyogenes* isolates were sent in agar transport tubes to University of Turku in larger batches. The year of isolation was provided. The strains were processed with an arbitrary study code and analysed similarly to isolates from HD1.

### Microbiological analysis and *emm* typing

Beta-haemolytic bacterial colonies were selected from the original throat culture plates and from isolates provided on transport tubes after reculturing on blood agar (TSA with sheep blood, BD). GAS isolates were confirmed with Lancefield antigen agglutination test (Remel^TM^ Streptex^TM^ Latex Group A, ThermoFisher). All isolates were *emm* typed using the CDC protocol [9].

For analysis, *emm* subtypes of the main *emm* types (*emm*1, *emm*4, *emm*12, *emm*28 and *emm*89) were grouped under the corresponding *emm* type (for example *emm*12.0 and *emm*12.37 were grouped into *emm*12). Due to the high prevalence of *emm*1.25 subtype, it was analysed separately and considered as an *emm* type in this study (Online Resource 1). The seven most common *emm* types (*emm*1, *emm*1.25, *emm*4, *emm*12, *emm*28, *emm*75 and *emm*89) were studied individually and the rest jointly under the group “others”.

In addition to *emm* types, isolates were analysed based on the *emm* cluster classification [13].

### Seasonality analysis

Seasonality analysis was performed only for isolates from HD1. The study period was divided into quarters representing the seasons: spring (March to May), summer (June to August), autumn (September to November) and winter (December to February). For the seasonality analysis, months after the onset of the COVID-19 pandemic (3/20–12/20) were excluded. The quarters of the remaining 2 years (3/18–2/20) were combined by season (6 months each). Seven most common *emm* types (*emm*1, *emm*1.25, *emm*4, *emm*12, *emm*28, *emm*75 and *emm*89) were studied individually and the rest jointly under the group “others”. In addition, seasonality was analysed on *emm* cluster level.

### Vaccine coverage analysis

The coverage of *emm* types of pharyngitis isolates collected in this study was evaluated in relation to composition of the 30-valent M-protein-based GAS vaccine candidates under development [14].

### Invasive GAS isolates

Clinical microbiological laboratories notify iGAS cases (isolations from blood and cerebrospinal fluid) and send the isolates to the National Infectious Disease Register (NIDR) maintained by the Finnish Institute of Health and Welfare (THL). THL performs *emm* typing for the isolates [9]. In this study, *emm-*type distribution data and year of isolation on all registered iGAS isolates in HD1 and HD2 covering January 2018–December 2020 were retrieved from NIDR.

### Statistical methods

Age and gender distribution analysis and seasonality analysis were only performed with the data from HD1. Due to asymmetrical distribution, median age was reported with range. For further analysis, age was categorized in 10-year age groups. Categorical data (*emm* type, gender, age group, season, months after onset of the COVID-19 pandemic) was summarized with counts (*n*) and percentages. Associations between categorical data were analyzed by the chi-square test. The prevalence of *emm* types in relation to patient age and gender was analyzed using binary logistic regression. First gender, age group and the interaction term between gender and age group were added to the multivariate models and from these models, non-significant factors were gradually omitted. Odds ratios (OR) with 95% Wald confidence intervals (95%CI) were reported. Because of the limited amount of data from iGAS isolates, only descriptive statistics were reported and it was not possible to carry out the association between seasonality and age and gender. All tests were performed as two-sided with a significance level set at 0.05. The analyses were carried out using SAS System, version 9.4 for Windows (SAS Institute Inc., Cary, NC, US).

## Results

**Fig. 1:**
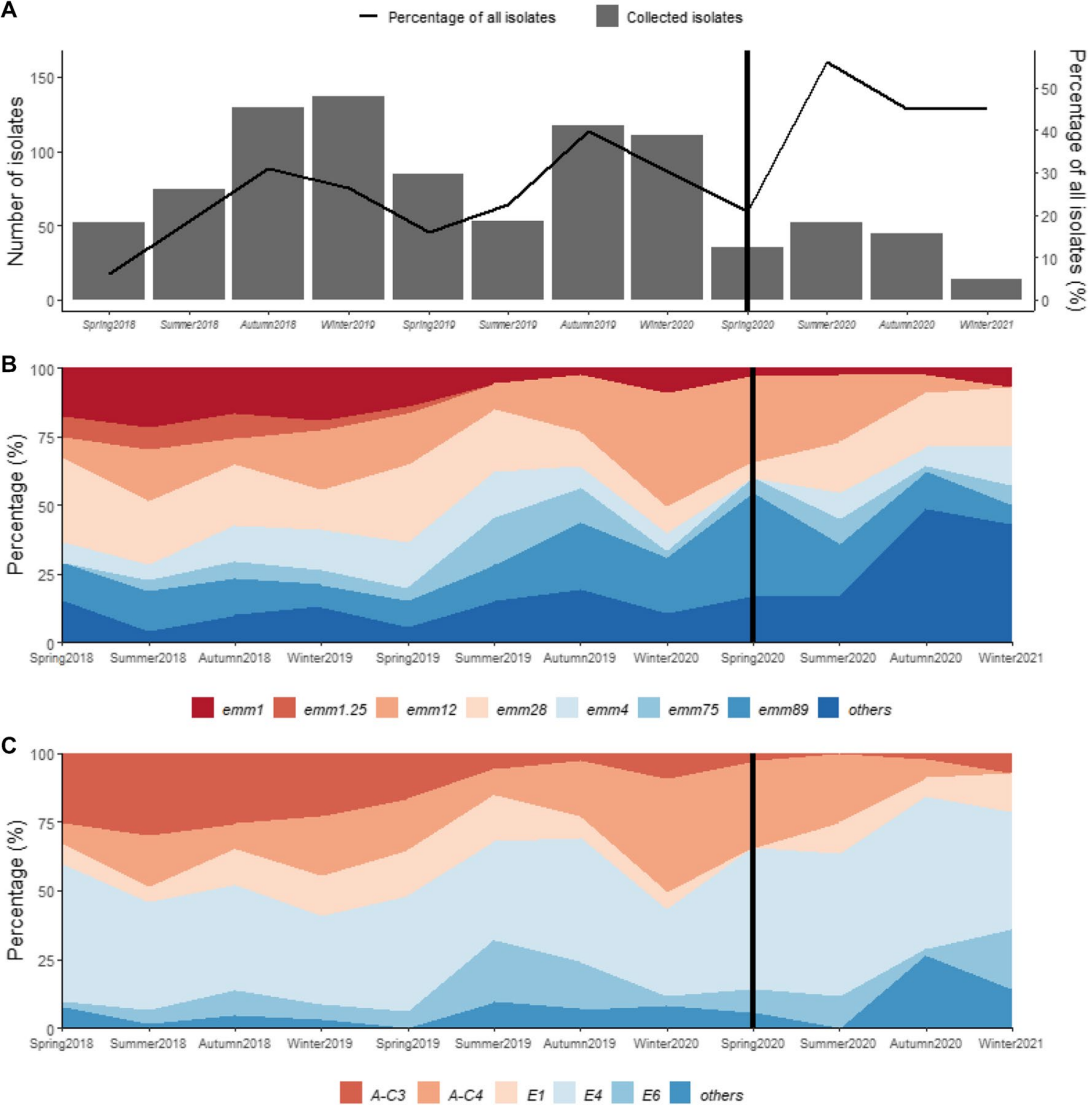
**A** The number of pharyngitis GAS isolates collected to our study by season from Hospital District 1 between March 2018 and December 2020 (bar chart) in relation to all GAS-positive pharyngitis findings from the same period collected in the same hospital district (line chart). Sample collection was halted for 6 weeks in Spring 2020 (16.3–26.4.2020). The vertical black bar marks the start of the COVID-19 pandemic (Spring 2020). Data is shown for each 3-month period; however, Winter 2021 includes only the month of December. The aim was to collect 10 isolates per week. The fulfilment was on average 7 isolates per week (range 0–19). **B** and **C** Seasonal fluctuations of main *emm* types (**B**) and *emm* clusters (**C**) among GAS pharyngitis isolates collected in Hospital District 1. Isolates collected after March 2020 (black bar) were not included in the seasonality analysis. *Significant difference (*p* < 0.05) observed for *emm*12, *emm*75, *emm*89 and the group ”others”

Overall, 1320 GAS isolates were collected during the 32-month study period, 904 from HD1 and 416 from HD2, respectively. This represents 14% of all GAS-positive pharyngitis cultures performed in these two laboratories during this period: 22% (of 4023 isolates) in HD1 and 7.4% (of all 5597 isolates) in HD2, respectively (Fig. 1 A).

### Hospital district 1

The median number of weekly collected strains was seven (range 0–19). Before the COVID-19 pandemic, the proportion of collected isolates in relation to all GAS isolates varied between 6.0 % (spring 2018, 52/865) and 40% (autumn 2019, 117/293). After the start of COVID-19 pandemic, the amount of isolates decreased, but the proportion remained high (37%, 146/392, Fig. 1 A).

**Fig. 2:**
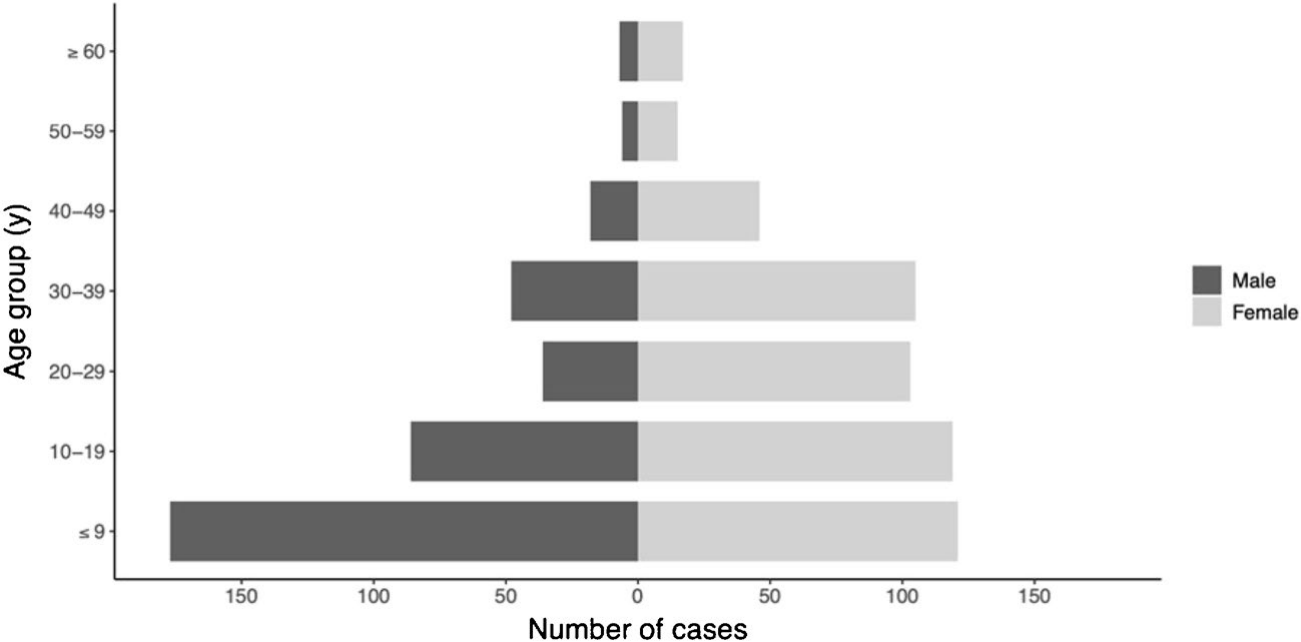
The age distribution in years (y) by gender, male (*n* = 378) and female (*n* = 526), of GAS pharyngitis cases in Hospital District 1 between March 2018 and December 2020. The age distribution was significantly different between genders (*p* < 0.0001)

The median age of patients was 17 years (range < 1–81 years). The most common age group was under 10-year-olds (298/904, 33% of all) and 71% of these were 5–9-year-olds (212/904, 23% of all). 58 % of the study subjects were female (526/904, 58%). The age distribution was significantly different between genders (*p* < 0.0001). Median age of males was 11 years (range 1–72 years), whereas within females it was 21 years (range < 1–81 years). Within males, most cases occurred in the age group under 10 years (177/378, 47%). Within females, the distribution was more uniform until age of 40 years (20–23% of the cases in each of the 10-year age group), whereas within males the distribution was skewed to right (Fig. 2).

**Fig. 3:**
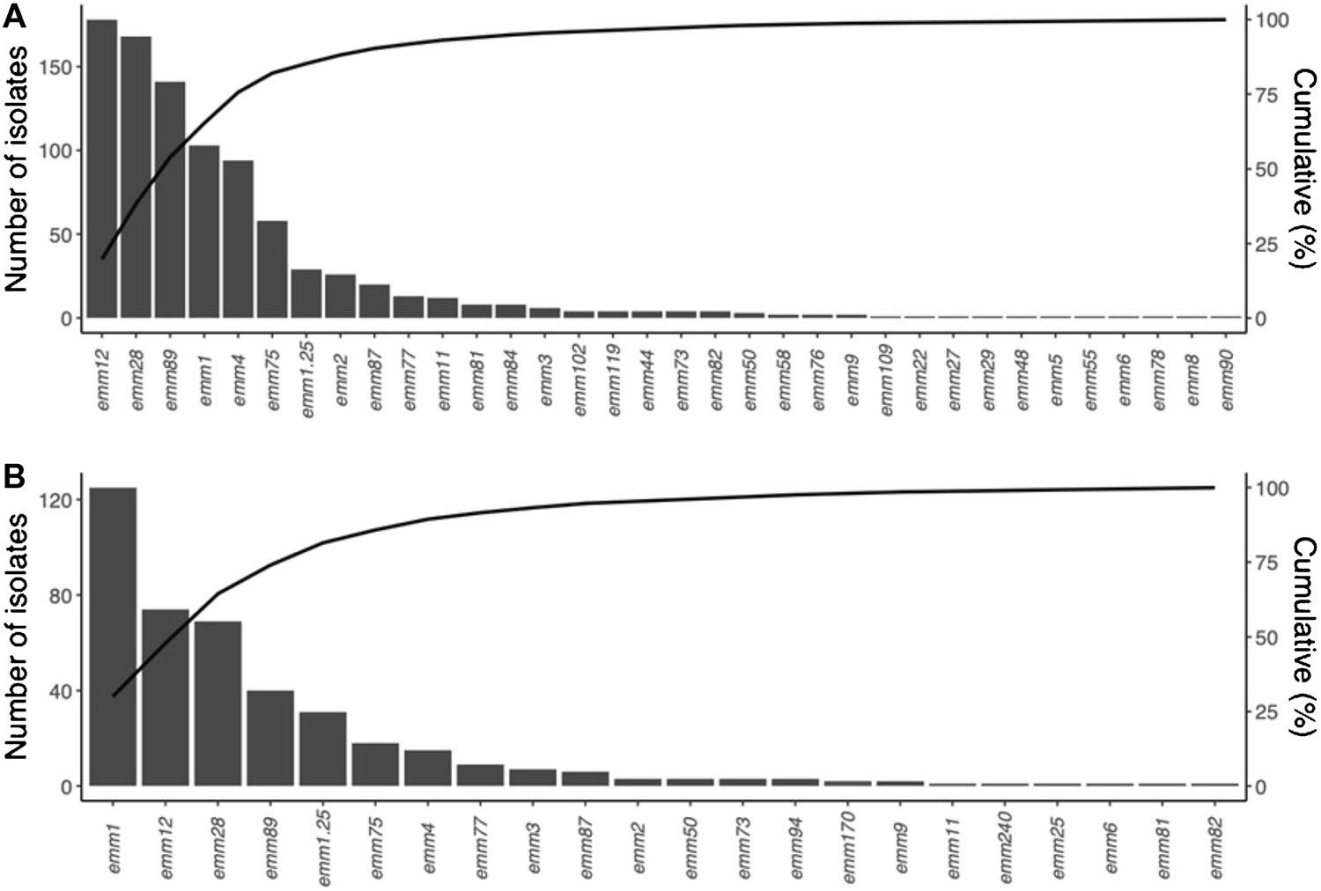
*Emm* types and cumulative percentage of GAS pharyngitis isolates collected **A** in Hospital District 1 (*n* = 904) and **B** in Hospital District 2 (*n* = 416) during the study period

Altogether, 34 different *emm* types were identified (Fig. 3 A, Online Resource 1). The four most common types were *emm*12 (20%), *emm*28 (19%), *emm*89 (16%) and *emm*1 (15%), and they covered 70% of the isolates. Two major *emm* subtypes dominated within *emm*1; *emm*1.0 (*n* = 96, 11% of all) and *emm*1.25 (*n* = 29, 3.2%), respectively, and within *emm*12; *emm*12.0 (*n* = 83, 9.2%) and *emm*12.37 (*n* = 63, 7.0%), respectively. Five of the most common *emm* cluster patterns were E4 (367/904 isolates, 41%), A-C4 (*n* = 178, 20%), A-C3 (*n* = 132, 15%), E1 (*n* = 95, 11%) and E6 (*n* = 79, 8.7%) (Online resorce 1).

97% of the isolates shared *emm* types putatively covered by the 30-valent GAS M-protein-based vaccine [14].

Age group was associated with prevalence of *emm*28 (*p* = 0.005) and *emm*89 (*p* = 0.028). *emm*28 was most common in the age group of 20–29 years. Compared to the rarest group among > 60 years old, the OR for the age group of 20–29 years is 7.0 (95%CI [2.5–19.6]). Also, all other age groups between 10 and 39 years old differed from the rarest age group (> 60 years old). *emm*89 was most common at the age group < 10 years; statistically significant difference was to all age groups between 20–49 years, ORs varying from 0.4 to 0.5 (Online Resource 2). No other statistical difference was observed between any of the identified *emm* type and age group or gender.

**Fig. 4:**
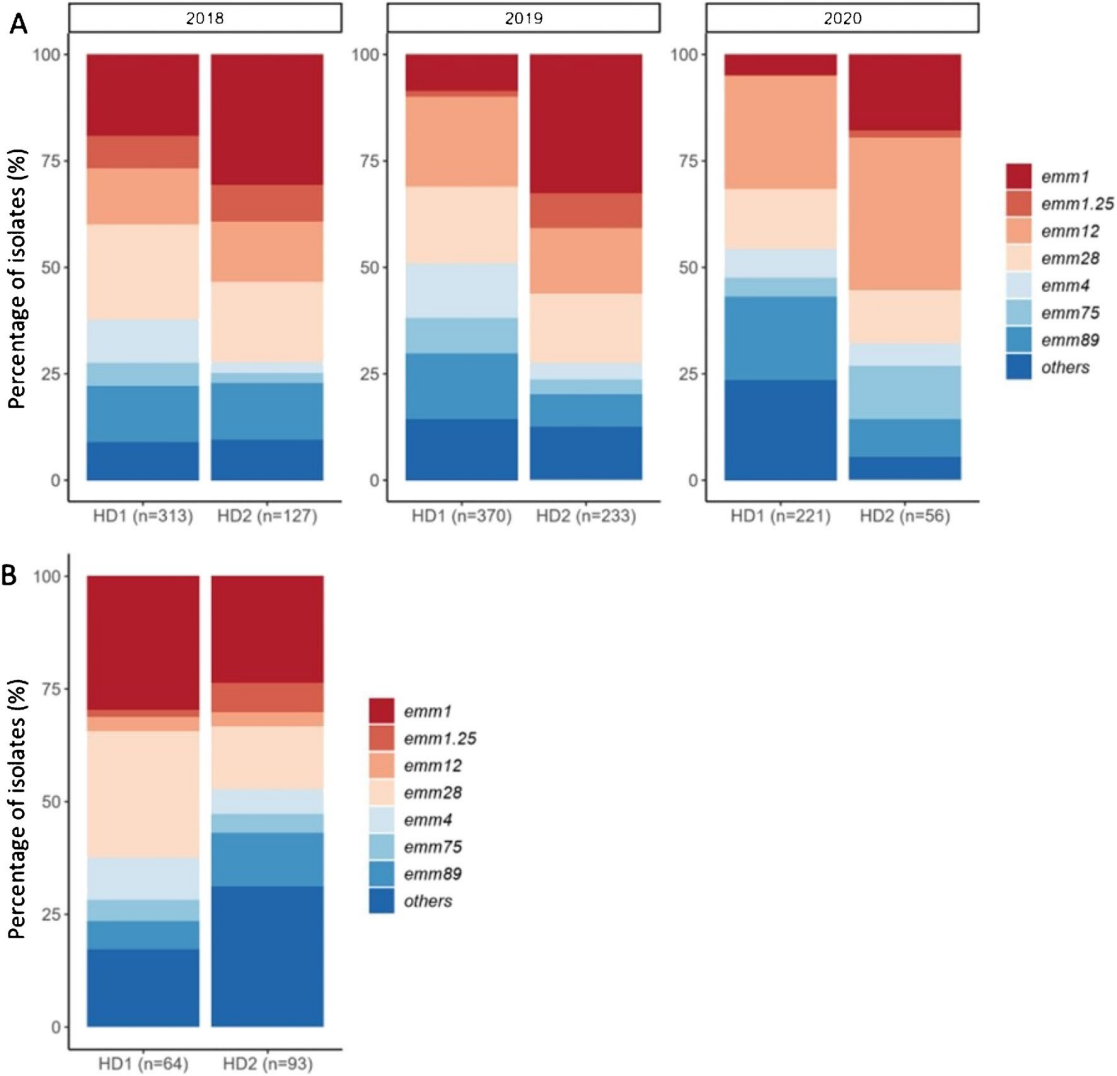
**A** Distribution of *emm* types among GAS pharyngitis isolates collected in Hospital District (HD) 1 (*n* = 904) and HD2 (*n* = 416) by year. The annual number of isolates is shown under each column. **B** Distribution of *emm* types of invasive GAS isolates reported from HD1 (*n* = 64) and HD2 (*n* = 93) during 2018–2020. The total number of isolates is shown under the column. In group “others” in iGAS isolates, the proportion of *emm*77 isolates was high in HD2 (13% of the isolates) and *emm*84 in HD1 (7.8%). Seven most common *emm* types (*emm*1, *emm*1.25, *emm*4, *emm*12, *emm*28, *emm*75 and *emm*89) were studied individually and the rest jointly under the group “others”

The *emm*-type distributions varied over time (Fig. 4). Frequency of *emm*1 decreased during the study (*n* = 60 (58%) in 2018, *n* = 11 (11%) in 2020), whereas *emm*12 increased (*n* = 41 (23%) vs *n* = 59 (33%)), respectively.

The number of isolates varied significantly in relation to season (*p* < 0.001, Fig. 1 A). Most isolates were collected during autumn (*n* = 246, 27% of all) and winter (*n* = 248, 27%). As to *emm* types, seasonality was noted to be significant within *emm*12 (*p* < 0.0001), *emm*75 (*p* = 0.003), *emm*89 (*p* = 0.003) and the group “others” (*p* = 0.0007) (Fig. 1 B). Most of the *emm*12 isolates were observed in winter (*n* = 76, 43%), *emm*75 in autumn (*n* = 23, 40%), *emm*89 in winter (*n* = 45, 32%) and “others” in autumn (*n* = 36, 38%). For *emm*1, *emm*1.25, *emm*4 and *emm*28, there was no statistically significant changes between seasons. Of note is the rise of the group “others” during the COVID-19 pandemic months.

### Hospital district 2

In total, 21 *emm* types were identified among the 416 isolates collected (Fig. 3 B, Online Resource 1). The four most common *emm* types were *emm*1 (38%), *emm*12 (18%), *emm*28 (17%) and *emm*89 (9.6%) covering 75% of the isolates. Two major *emm*1 subtypes dominated: *emm*1.0 (*n* = 119, 29%) and *emm*1.25 (*n* = 31, 7.5%). The annual distribution of *emm* types differed (Fig. 4A). *emm*1 decreased (*n* = 39 (31%) in 2018 vs *n* = 10 (18%) in 2020) and *emm*12 increased (*n* = 18 (14%) vs *n* = 20 (36%)), respectively. Five of the most common *emm* cluster patterns were A-C3 (*n* = 156, 38% of all), E4 (*n* = 124, 30%), A-C4 (*n* = 74, 18%), E6 (*n* = 23, 5.5%) and E1 (*n* = 15, 3.6%). One *emm* type (*emm240.3*) did not belong to any known *emm* cluster (Online resorce 1).

Ninety-seven percent of the isolates shared *emm* types putatively covered by the 30-valent GAS M-protein-based vaccine (14).

### *Emm *type distribution among iGAS cases

Altogether, 157 iGAS cases were reported (64 in HD1 and 93 in HD2) between 2018–2020. The four most common *emm* types in HD1 were *emm*1 (30%), *emm*28 (28%), *emm*4 (9.3%) and *emm*84 (7.8%) and in HD2 *emm*1 (24%), *emm*28 (14%), *emm*77 (13%) and *emm*89 (12%) (Fig. 4 B). Compared by source of specimen, *emm*12 was found to be more common among pharyngitis than iGAS isolates in both hospital districts (Figs. 3 and 4 B). While *emm*77 and *emm*84 were common in iGAS, they were rare among pharyngitis isolates (grouped to “others”).

## Discussion

This study describes the epidemiology of group A streptococcal pharyngitis in two hospital districts in Southern Finland covering approximately one million inhabitants. Systematic, prospective collection allowed to study variation in the *emm*-type distribution and the seasonality.

From the HD1, a clear difference was observed in the age distributions and prevalence between the genders. In females, the cases occurred more uniformly until the age of 40, whereas in males most cases were in early childhood. Similar observations have recently been reported from a retrospective, register-based study from Canada [15]. The reasons behind these differences remain unknown and can only be speculated. Social and occupational factors may also affect the findings such as contacts with children in general. Our observation that young boys were overrepresented is worth further investigation; the distribution of GAS pharyngitis between genders in relation to age is not often studied. Asymptomatic carriage of GAS has been reported to be over 10% for children over 5 years of age [16, 17], which may reflect the higher disease burden as well. In our study, most of the isolates were collected from 5 to 9-year-olds.

In Western countries, same *emm* types such as *emm*1, *emm*89 and *emm*28, have been observed to associate with both iGAS and pharyngitis [8, 12, 17–22]. The same was noticed also in our study.

Interestingly, *emm*28 was found to be common within 20–29-year-olds. Noteworthy, *emm28* has previously been associated to iGAS infections in fertile aged women and puerperal sepsis [18, 23]. No statistical differences between gender and age groups were observed in this study, which might be due to the small number of cases per group.

The finding that *emm*89 was more common in children under 10 years of age, is new, but not unexpected. A new acapsular clone of *emm*89 emerged in the mid-2010s in many countries, including Finland [24]. This clone has an advantage in persistence and transmission also among pharyngitis cases [25, 26].

Most of the isolates were cultured during autumn and winter, which is supported by previous studies [15, 27]. Interestingly, the prevalence of *emm*1, *emm*1.25, *emm*4 and *emm*28 were not affected by season. The dominance of *emm1* in our collection might reflect to the contemporary *emm*1 iGAS epidemic in Finland [28]. Likewise, the disappearance of *emm*1.25 from both hospital districts by 2020 may reflect the same. Similarly, *emm*4 and *emm*28 have been associated with epidemic behaviour [21].

Two hospital districts were included to broaden the epidemiological and geographical coverage of the study. The major difference observed was that *emm*1 dominated only in HD2. An *emm*1 iGAS epidemic occurred in HD2 in 2019, whereas in HD1 just before our study [29]. Interestingly, the *emm *type distributions in these two districts varied also between the pharyngitis and iGAS isolates. This underlies the fact that regional epidemiology may vary also within relatively short distances (*c.* 160 kilometres in between).

Due to overall high morbidity of GAS infections, a vaccine against GAS would be important. In our study, the putative coverage of the M-protein-based GAS vaccines would be over 97%, which is in line with other studies [3, 10, 21]. Due to the cross protection between *emm* types, the coverage might even be wider [11]. However, regional differences in *emm* distribution occur, which complicate putative vaccination strategies [10].

The study period included the start of the COVID-19 pandemic, which changed the epidemiology of GAS pharyngitis. The number of GAS-positive pharyngitis cultures decreased, and the *emm *type distribution diversified with previously less common *emm* types arising. As however the proportion of GAS pharyngitis isolates included into our study during these months remained high, we find this observation of interest. A similar switch in *emm* types has been reported within the iGAS cases in Finland and elsewhere [8, 28, 30]. Recently, a surge of GAS infections has been noted in many countries, probably linked to a higher proportion of individuals susceptible to these infections due to less exposure to GAS as result of pandemic lockdown measures [6, 7].

Our study has some limitations. The study protocol aimed to collect a scientifically representative set of GAS pharyngitis isolates within a certain time frame. We acknowledge that the study material covers only part of all culture positive GAS findings in the respective clinical laboratories. Cultures were collected from GAS-positive pharyngitis patients, but GAS carriers suffering from viral pharyngitis may have though been included. Our collection may include multiple isolates from one individual, as these could not be excluded during the collection process. The collection procedure differed slightly between the hospital districts, which limited the analysis of seasonality and patient characteristics to include only HD1. Lastly, we acknowledge that the clinical microbiological laboratories which collected the GAS isolates serve mainly the public health care system leaving private sector and occupational health care neglected.

## Conclusions

The prevalence of GAS pharyngitis was different between genders, particularly in different age groups. Females were overrepresented, whereas in young children, males were clearly dominating. Seasonal fluctuation was observed, but some *emm* types behaved more epidemically. *emm*28 was more common in the age group of 20–29-year-olds, and *emm*89 in under 10-year-olds. COVID-19 pandemic changed the epidemiology of GAS pharyngitis resulting in a wider spectrum of *emm* types among the fewer strains cultured.

### Supplementary information

ESM 1 Online Resource 1. *Emm* types and *emm* clusters of pharyngitis GAS isolates collected in Hospital District (HD) 1 (n=904) and HD2 (n=416) during the study period. (DOCX 31 kb)

ESM 2 Online Resource 2. The prevalence of *emm*28 and *emm*89 in different age groups, corresponding odds ratios (OR) and 95% confidence intervals (CI). (DOCX 13 kb)

## Data Availability

The datasets generated during the current study are not publicly available as they contain health related data but limited datasets (without any identifiable, person-related data) are available from the corresponding author on reasonable request.
