# Supplementary material for: Epidemiology and emm types among group A streptococcal pharyngitis in Finland: a prospective laboratory-based study
Source: Eur J Clin Microbiol Infect Dis. 2023 Nov 27;43(2):233–41. doi: 10.1007/s10096-023-04714-6 (PMC10821968; doi:10.1007/s10096-023-04714-6)
Supplement: Supplementary file 1 — Online Resource 1. Emm types and emm clusters of pharyngitis GAS isolates collected in Hospital District (HD) 1 (n=904) and HD2 (n=416) during the study period. (DOCX 31 kb) [file 10096_2023_4714_MOESM1_ESM.docx]

Online resource 1. *Emm* types and *emm* clusters of pharyngitis GAS isolates collected in Hospital District (HD) (n=904) and HD2 (n=416) during the study period.

**HD1 (n=904):**

**HD2 (n=416):**
