# Supplementary material for: Epidemiology and emm types among group A streptococcal pharyngitis in Finland: a prospective laboratory-based study
Source: Eur J Clin Microbiol Infect Dis. 2023 Nov 27;43(2):233–41. doi: 10.1007/s10096-023-04714-6 (PMC10821968; doi:10.1007/s10096-023-04714-6)
Supplement: Supplementary file 2 — Online Resource 2. The prevalence of emm28 and emm89 in different age groups, corresponding odds ratios (OR) and 95% confidence intervals (CI). (DOCX 13 kb) [file 10096_2023_4714_MOESM2_ESM.docx]

**Online resource 2**: The prevalence of *emm28* and *emm89* in different age groups, corresponding odds ratios (OR) and 95% confidence intervals (CI). *Statistically significant difference

A) *emm28* age groups compared to rarest age group over 60 years olds (n=9)

| Age group (n) | OR | 95%CI |
| --- | --- | --- |
| Under 10 years old (68) | 2.0 | 0.9-4.8 |
| 10-19 years old (34) | 3.0* | 1.2-7.5 |
| 20-29 years old (11) | 7.0* | 2.5-19.6 |
| 30-39 years old (29) | 2.6* | 1.0-6.4 |
| 40-49 years old (13) | 2.4 | 0.8-6.5 |
| 50-59 years old (4) | 2.6 | 0.7-10.0 |

B) *emm89* age groups compared to most common age group under 10 years olds (n=32)

| Age group (n) | OR | 95%CI |
| --- | --- | --- |
| 10-19 years old (27) | 1.3 | 0.7-2.2 |
| 20-29 years old (30) | 0.4* | 0.3-0.8 |
| 30-39 years old (28) | 0.5* | 0.3-0.9 |
| 40-49 years old (15) | 0.4* | 0.2-0.8 |
| 50-59 years old (4) | 0.5 | 0.2-1.7 |
| Over 60 years old (5) | 0.5 | 0.2-1.3 |
